# The triglyceride synthesis enzymes DGAT1 and DGAT2 have distinct and overlapping functions in adipocytes[S]

**DOI:** 10.1194/jlr.M093112

**Published:** 2019-04-01

**Authors:** Chandramohan Chitraju, Tobias C. Walther, Robert V. Farese

**Affiliations:** Department of Genetics and Complex Diseases,* Harvard T.H. Chan School of Public Health, Boston, MA 02115; Department of Cell Biology,† Harvard Medical School, Boston, MA 02115; Broad Institute of MIT and Harvard,§ Cambridge, MA 02142; Howard Hughes Medical Institute,** Boston, MA 02115

**Keywords:** adipose tissue, diacylglycerol acyltransferase, high-fat diet, induced obesity, glucose intolerance, endoplasmic reticulum stress

## Abstract

Mammals store metabolic energy as triacylglycerols (TGs) in adipose tissue. TG synthesis is catalyzed by the evolutionarily unrelated acyl-CoA:diacylglycerol acyltransferase (DGAT) enzymes DGAT1 and DGAT2, which catalyze the same reaction and account for nearly all TG synthesis. The reasons for their convergent evolution to synthesize TGs remain unclear. Mice lacking DGAT1 are viable with reduced fat stores of TGs, whereas DGAT2 KO mice die postnatally just after birth with >90% reduction of TGs, suggesting that DGAT2 is the predominant enzyme for TG storage. To better understand the functional differences between the DGATs, we studied mice fed chow or high-fat diets lacking either enzyme in adipose tissue. Unexpectedly, mice lacking DGAT2 in adipocytes have normal TG storage and glucose metabolism on regular or high-fat diets, indicating DGAT2 is not essential for fat storage. In contrast, mice lacking DGAT1 in adipocytes have normal TG storage on a chow diet but moderately decreased body fat accompanied by glucose intolerance when challenged with a high-fat diet. The latter changes were associated with the activation of ER stress pathways. We conclude that DGAT1 and DGAT2 can largely compensate for each other for TG storage but that DGAT1 uniquely has an important role in protecting the ER from the lipotoxic effects of high-fat diets.

Highly reduced carbon molecules, such as triacylglycerols (TGs), are the major form of metabolic energy stores in eukaryotic cells and organisms. TG phases separate within cells to form lipid droplets (LDs) (1–3) that exclude water and, as a consequence, provide energy-dense depots of reduced carbons. LDs can be formed in many different cell types, but for most vertebrates, the majority of TG stores are found in the LDs of adipocytes. In mammals, adipocytes are found in different adipose tissues throughout the body and include subcutaneous or visceral collections of white or brown adipocytes. White adipocytes are particularly well adapted to store TGs inasmuch as they predominantly have a “unilocular” LD that occupies the majority of the cell volume (4, 5).

The mechanisms that determine the capacity of adipocytes to store TGs are not well understood. In modern societies, humans are often confronted with an oversupply of metabolic energy, leading to excessive TG storage in adipocytes and obesity, which has become a worldwide epidemic (6). In obesity, the capacity for TG storage in adipocytes can be overwhelmed, leading to adipocyte and adipose tissue dysfunction, and by the accumulation of excess lipids in nonadipose tissues, resulting in tissue dysfunction known as lipotoxicity (7, 8). These pathological processes are thought to underlie and contribute to metabolic consequences of obesity, such as hepatic steatosis and type 2 diabetes (9–11).

In eukaryotes, TG synthesis is catalyzed by the acyl-CoA:diacylglycerol acyltransferase (DGAT) enzymes DGAT1 or DGAT2 (12–14). Both catalyze the same reaction, condensing diacylglycerol and fatty acyl-CoA to form TGs, but they are evolutionarily unrelated (15, 16). DGAT1 and DGAT2 account for the vast majority of TG synthesis in cultured adipocytes (17, 18). A longstanding question is how the two fundamentally different enzymes contribute to TG storage in adipose tissue. DGAT1 is a member of the membrane-bound *O*-acyltransferase gene family (15) and is a multitopic ER membrane protein, with its likely active site on the luminal side of the ER membrane (19–23). It can transfer a fatty acyl moiety to multiple acceptors in addition to diacylglycerol, including retinol or long-chain alcohols, to form retinyl esters or waxes, respectively (15, 24). In contrast, DGAT2 activity is largely specific to the synthesis of TGs, although acylceramide synthesis activity has been reported (25) The DGAT2 protein differs from DGAT1 in that it has a single membrane-embedded hairpin with its catalytic domain on the cytoplasmatic side of the ER membrane (26). DGAT2 can also relocalize around LDs, where it appears to be required for LD-localized TG synthesis (26–28). Because DGAT2 has an apparently lower *K_m_* for substrates than DGAT1 (13), it has been suggested that it esterifies substrates at lower concentrations for the storage of TGs, whereas DGAT1 may only operate at higher substrate concentrations (13).

KO mice deficient for either DGAT enzyme have revealed striking differences in the physiological functions of the enzymes. Mice lacking DGAT1 are viable and metabolically healthy, with ∼50% decreased TG stores in adipose tissue (29). These mice have increased energy expenditure, are resistant to diet-induced obesity and glucose intolerance (30, 31), and live ∼25% longer than wild-type mice (32). This complex phenotype likely involves cross-talk among different organs, including the intestine, liver, and adipose tissue (15, 29). By analyzing mice lacking DGAT1 specifically in adipocytes, we recently found that DGAT1-mediated TG synthesis functions not only in energy storage but also in protecting adipocytes from lipotoxicity during lipolysis (e.g., when fatty acids reach high intracellular concentrations as TGs are rapidly hydrolyzed) (18).

In marked contrast to DGAT1 KO mice, mice lacking DGAT2 die shortly after birth, presumably due to impaired skin permeability with increased transepidermal water loss, which leads to rapid dehydration and death (33). Importantly, DGAT2 KO mice have dramatically reduced TG storage (with less than 10% of the normal levels), which led to the hypothesis that DGAT2 is primarily responsible for mammalian TG storage (15, 33). However, due to the vastly different phenotypes of the global KO models, including one perinatally lethal model, this hypothesis has not been rigorously tested.

To determine the functional roles of DGAT1 versus DGAT2 in TG metabolism, we generated mice lacking DGAT2 specifically in adipose tissue and compared TG synthesis and storage under basal and high-fat diet (HFD) feeding conditions in mice lacking either DGAT1 or DGAT2 in adipose tissue (designated ADGAT1 or ADGAT2 KO mice). Unexpectedly, we found that ADGAT2 KO mice have normal TG depots and that the two enzymes can largely compensate for TG storage under basal conditions. However, important differences emerge when the mice are fed an HFD. In this case, ADGAT1 KO mice store TGs less efficiently and exhibit signs of lipotoxicity, shedding light on the functional differences between the two enzymes.

## MATERIALS AND METHODS

### Targeting vector and ES cells

**Fig. 1.**
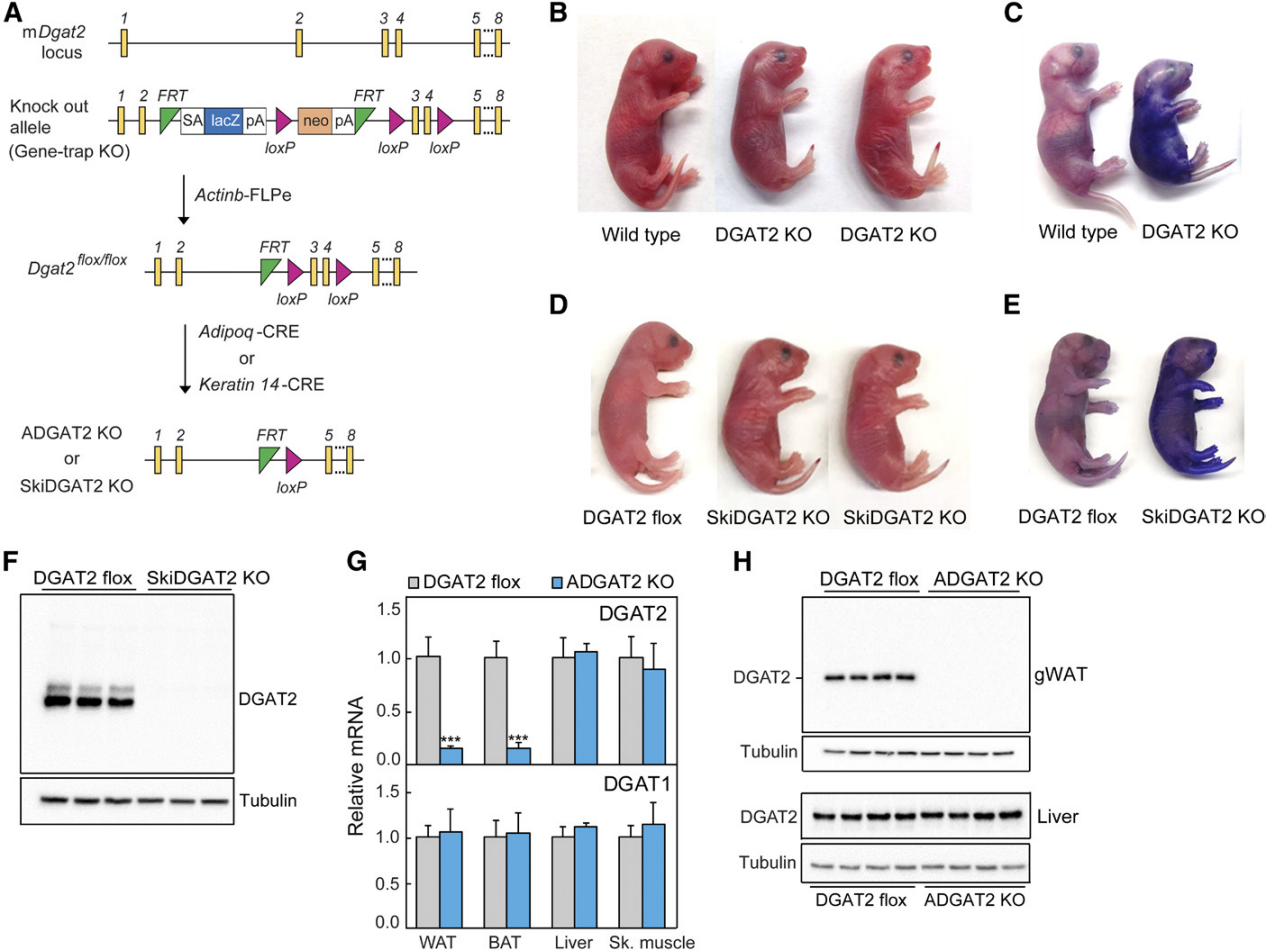
DGAT2 and SkiDGAT2 KO mice die shortly after birth. A: Strategy for generating DGAT2 KO, DGAT2 flox mice, and tissue-specific DGAT2 KO mice. A “knock-out first/conditional-ready” gene-targeting vector was used to generate targeted ES cells. The gene-trap cassette is located between two FRT sites. B, C: DGAT2 KO mice die within a few hours after birth due to defective skin-barrier function. D, E: SkiDGAT2 KO mice were generated by crossing DGAT2 flox mice with the mice expressing Cre recombinase under the control of the human keratin-14 promoter. SkiDGAT2 KO mice die within a few hours after birth due to defective skin-barrier function. F: The DGAT2 protein is absent in the skin of SkiDGAT2 KO mice (*n* = 3). G: Adipose tissue-specific ADGAT2 KO were generated by crossing DGAT2 flox mice with the mice expressing Cre recombinase under the control of the mouse adiponectin promoter. *Dgat1* and *Dgat2* mRNA levels in gWAT of DGAT2 flox and ADGAT2 KO mice (*n* = 5). H: The DGAT2 protein is absent in gWAT of ADGAT2 KO mice (*n* = 4). Data are presented as mean ± SD. ***P < 0.001.

Targeted embryonic stem (ES) cells were obtained from the European Conditional Mouse Mutagenesis Program (EUCOMM). The targeting vector contains the L1L2_Bact_P cassette, which is composed of an FRT site (after the 5′ arm, which contains the *Dgat2* exon 2), followed by a lacZ sequence and loxP site. This first loxP site is followed by neomycin phosphotransferase II sequence under the control of the human β-actin promoter, an SV40 polyA, and a second FRT and loxP site, respectively (34) (**Fig. 1A**, supplemental Fig. S1B) (before *Dgat2* exon 3). A third loxP site is inserted downstream of the targeted exons (*Dgat2* exon 4). The critical exons (exon 3 and 4) are thus flanked by loxP sites. A “conditional-ready” (floxed) allele can be created by flp recombinase expression in mice carrying this allele. Subsequent Cre expression results in a KO mouse. If Cre expression occurs without flp expression, a reporter KO mouse will be created. The vector was electroporated into the JM8A1.N3 ES cell line from C57BL/6N mice (35).

### ES cell culture, blastocyst injections, and transfer to foster mothers

We obtained two different ES cell clones from EUCOMM. ES cells were thawed and then cultured on mitomycin C–treated feeder cells (STO feeder cell line) per EUCOMM protocols. The ES cell-culture medium contained 500 ml KO DMEM (Gibco), 90 ml lot-tested FBS (Gibco), 5 ml 100× l-glutamine (Gibco), 5 μl 100 β-mercaptoethanol (Sigma-Aldrich; 360 μl/500 ml 100× sterile-filtered PBS stored at −20°C), 100 μg/ml G418 (Gibco), and ESGRO leukemia inhibitory factor supplement (Chemicon). Total DNA from ES cells was isolated and tested by PCR for neomycin phosphotransferase II to confirm the presence of the targeted vector. For blastocyst injections, ES cells were trypsinized when they were between 50% and 70% confluence (supplemental Fig. S1A) per EUCOMM protocol and submitted to Gladstone Institutes for blastocyst injections and transfer to foster mothers.

### Generation of DGAT2 KO and DGAT2 flox, ADGAT2 KO, and SkiDGAT2 KO mice

Founders (chimeras) were identified by PCR for neomycin phosphotransferase II (supplemental Fig. S1A). The transmission of the mutant allele was confirmed by crossing founders with C57BL/6J mice. DGAT2 global KO mice were generated by crossing the mice heterozygous for the gene-trap allele. To generate *Dgat2*^flox/flox^ (DGAT2 flox) mice, heterozygous mice for the gene-trap allele were crossed with transgenic mice expressing FLP-e recombinase under the control of the human β-actin promoter (supplemental Fig. S1D) (36). The deletion of the gene-trap cassette and orientation of loxP sites were confirmed by PCR. ADGAT2 KO mice were generated by crossing DGAT2 flox mice with transgenic mice expressing Cre recombinase under the control of the mouse adiponectin promoter (37). We generated skin-specific *Dgat2* (SkiDGAT2) KO mice by crossing DGAT2 flox mice with transgenic mice expressing Cre recombinase under the control of the human keratin-14 promoter (38).

### Mouse husbandry and dietary intervention studies

All animal experiments were performed under the guidelines established by the Harvard Center for Comparative Medicine. Mice were maintained in a barrier facility, at normal room temperatures, on a regular 12 h light and dark cycle and had ad libitum access to food and water unless otherwise stated. Mice were fed a standard laboratory chow diet (PicoLab Rodent Diet 20 5053) or a Western-type HFD (Envigo TD.88137).

### Mouse DGAT1 and DGAT2 antibody generation

We custom-generated rabbit polyclonal antibodies against mouse DGAT1 and DGAT2 (GenScript). To generate DGAT1 antibodies, we used an N-terminal peptide corresponding to amino acids 23–52 (NH2-GGSGPKVEEDEVR DAAVSPDLGAGGDAPAP-COOH) of DGAT1 as the antigen. The peptide was conjugated to mcKLH with the Imject EDC mcKLH spin kit (Thermo Scientific) according to the manual. The antigen was then injected into rabbits to produce the antibody. We injected five rabbits, and two gave a better immune response. To generate DGAT2 antibodies, we used a C-terminal peptide corresponding to amino acids 373–388 (NH2-KTKFGLPETEVLEVN-COOH) of DGAT2 as the antigen. We injected five rabbits, and two gave an appropriate antibody. Antibodies were affinity-purified and used at a 1:1,000 dilution in 5% milk in TBST and incubated overnight at 4°C.

### DGAT activity assay

DGAT enzymatic activities were measured in membrane fractions isolated from white adipose tissue (WAT) of DGAT2 flox and ADGAT2 KO mice. Enzymatic activities were measured at *V_max_* substrate concentrations. The assay mixture contained 5–10 µg membrane proteins; 100 µM 1,2-dioleoyl-sn-glycerol; 25 µM oleoyl-CoA, which contained [^14^C]oleoyl-CoA as the tracer; and 25 mM MgCl_2_ for the DGAT1 assay and 1 mM MgCl_2_ for the DGAT2 assay in an assay in buffer containing 100 mM Tris-HCl (pH 7.4) and protease inhibitors. Using 25 mM MgCl_2_ or greater in the assay renders it largely specific for DGAT1 in adipocytes (13, 18). The reaction was carried out as described earlier (18). After stopping the reaction, lipids were extracted and then separated by TLC using a hexane/diethyl ether/acetic acid (80:20:1) solvent system. The TLC plates were exposed to a phosphor imager screen and developed. TLC plates were exposed to a phosphor-imaging cassette overnight and revealed by the Typhoon FLA 7000 phosphor imager.

### RNA extraction and qPCR

Total RNA was isolated using the RNeasy Kit (Qiagen) according to the manufacturer’s instructions. For isolating RNA from WAT, an RNeasy Lipid Tissue (Qiagen) was used. cDNA was synthesized using the iScript cDNA Synthesis Kit (Bio-Rad), and quantitative PCR (qPCR) was performed in triplicates using the SYBR Green PCR Master Mix Kit (Applied Biosystems).

### Tissue lipid analysis

Liver and skeletal muscle were homogenized in 1 ml lysis buffer (0.25 M sucrose, 50 mM Tris HCl, pH 7.0, with protease inhibitor cocktail) using a Misonix Sonicator 4000. The homogenate was mixed with 5 ml chloroform-methanol (3:2 v/v) and extracted overnight by rotation. Upon centrifugation at 3,000 *g* at room temperature for 10 min, 100 µl of the lower organic phase was collected and dried in a speed vacuum. To the dried lipids, 100–300 µl 0.1% Triton X-100 was added and sonicated. TGs and total cholesterol were measured using Infinity TM Triglycerides reagent (Thermo Fisher Scientific) and a cholesterol E kit (Wako Diagnostics), respectively, according to manufacturer’s protocol. For plasma lipid measurements, 5 µl plasma was used directly.

### Immunoblotting

Cells were lysed using RIPA lysis buffer. Tissues were lysed in buffer containing 250 mM sucrose, 100 mM Tris-HCl (pH 7.4), and protease inhibitors in a Dounce homogenizer. Proteins were denatured in Laemmli buffer, separated on 10% SDS-PAGE gel, and transferred to a PVDF membrane (Bio-Rad). Membranes were blocked with blocking buffer for 2 h in TBST containing 5% BSA or 5% milk and then incubated with primary antibodies overnight. The membranes were then washed three times with TBST for 10 min and incubated in mouse secondary antibodies (Santa Cruz Biotechnology) at 1:5,000 dilutions in blocking buffer. Membranes were washed again three times with TBST for 10 min and revealed using the Super Signal West Pico kit (Thermo Fisher Scientific). DGAT1 antibodies were a gift from Jin Ye (UT Southwestern Medical Center). CHOP and Bip antibodies were purchased from Cell Signaling Technology.

### Statistical analyses

Data are presented as mean ± SD or mean ± SEM. Statistical significance was evaluated by unpaired two-tailed Student’s *t*-test or two-way ANOVA with repeated measures.

## RESULTS

### Mice lacking DGAT2 in the epidermis die postnatally, but mice lacking DGAT2 in adipose tissue are viable

To analyze the contribution of DGAT2 to TG synthesis in different cell types, we generated tissue-specific KOs of DGAT2 by using a gene-trap allele that allows for the generation of either whole-body or tissue-specific KO mice (34) (Fig. 1A). We first generated DGAT2 KO mice by intercrossing mice that were heterozygous for the targeted gene-trap allele (Fig. 1A). Consistent with our previous findings (33), DGAT2 KO mice generated by this allele died shortly after birth and had an apparent defect in the epidermal water-permeability barrier (Fig. 1B, C), including increased uptake of toluidine blue (a water-based dye). These findings validate that the gene-trap allele correctly targeted and inactivated *Dgat2*.

Previous studies did not resolve whether the skin abnormalities of DGAT2 KO mice resulted from the loss of DGAT2 function in the skin or other tissues (33). We therefore generated SkiDGAT2 KO mice by crossing DGAT2 flox mice with transgenic mice expressing Cre recombinase under the control of the human keratin-14 promoter (38) (Fig. 1A). SkiDGAT2 KO mice were markedly abnormal in appearance and died shortly after birth, essentially phenocopying the skin permeability-barrier defect of DGAT2 KO mice (Fig. 1D, E). Western blot analyses confirmed that DGAT2 was absent in the epidermis of SkiDGAT2 KO mice (Fig. 1F). These findings indicate that DGAT2 has an essential and tissue-autonomous function in maintaining skin lipids important for the epidermal water barrier in mice.

We next generated ADGAT2 KO mice by crossing DGAT2 flox mice with transgenic mice expressing Cre recombinase under the control of the murine adiponectin promoter (Fig. 1A) (37). In contrast to the SkiDGAT2 KO mice, ADGAT2 KO mice were viable and appeared healthy, with no apparent defects in their skin. Real-time qPCR analysis showed *Dgat2* mRNA levels were decreased by ∼85% in gonadal WAT (gWAT) and brown adipose tissue (BAT) of ADGAT2 KO mice but were unchanged in livers and skeletal muscle (Fig. 1G). We found no evidence for compensatory upregulation of DGAT1 mRNA expression in gWAT and BAT of ADGAT2 KO mice (Fig. 1G). Western blot analyses showed a complete lack of DGAT2 protein in gWAT of ADGAT2 KO mice, confirming complete KO of DGAT2 in adipose tissue (Fig. 1H).

### DGAT1 and DGAT2 are functionally redundant for TG storage in adipocytes under basal conditions

**Fig. 2.**
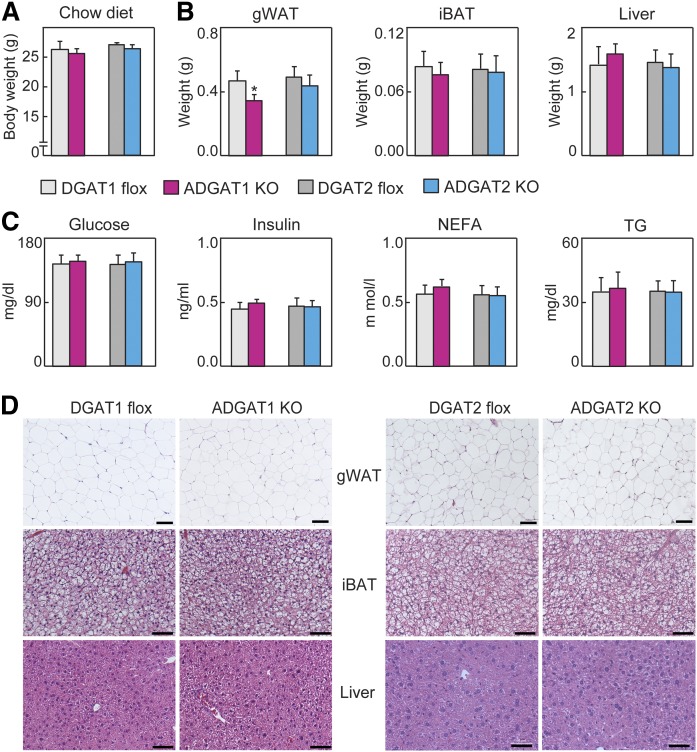
DGAT1 and DGAT2 largely compensate for TG storage in adipose tissue in the chow-fed state. A: Body weights of mice fed a chow diet (*n* = 10–12). B: Organ weights of mice fed a chow diet (*n* = 8 per genotype). C: Plasma parameters of mice fed a chow diet (*n* = 7 per genotype). D: H&E-stained sections of gWAT, iBAT, and livers of mice fed a chow diet (representative images of eight mice per genotype). Scale bars: 50 µm. Data are presented as mean ± SD. *P < 0.05 by *t*-test. iBAT, intrascapular brown adipose tissue; NEFA, nonesterified fatty acid.

Based on the dramatic reduction of TG levels in animals lacking DGAT2 globally (33), we expected that ADGAT2 KO mice would have markedly reduced TG levels in adipose tissue, possibly even resulting in lipodystrophy. Unexpectedly, we found that ADGAT2 KO mice fed a chow diet had normal body weight and fat depots (**Fig. 2A**, B). Moreover, plasma levels of glucose, insulin, nonesterified fatty acids, and TGs were similar in ADGAT2 KO mice and DGAT2 flox (control) mice fed a chow diet (Fig. 2C). Additionally, WAT, BAT, and the liver all had normal histological appearances in ADGAT2 KO mice (Fig. 2D). Thus, unexpectedly, ADGAT2 KO mice had normal TG storage with chow feeding. Under these conditions, ADGAT1 KO mice had a virtually identical phenotype to ADGAT2 KO mice, with the exception of a modest 20% reduction in gonadal fat weight (Fig. 2B).

### Reduced TG storage in fat and impaired glucose tolerance in ADGAT1 KO mice fed an HFD

**Fig. 3.**
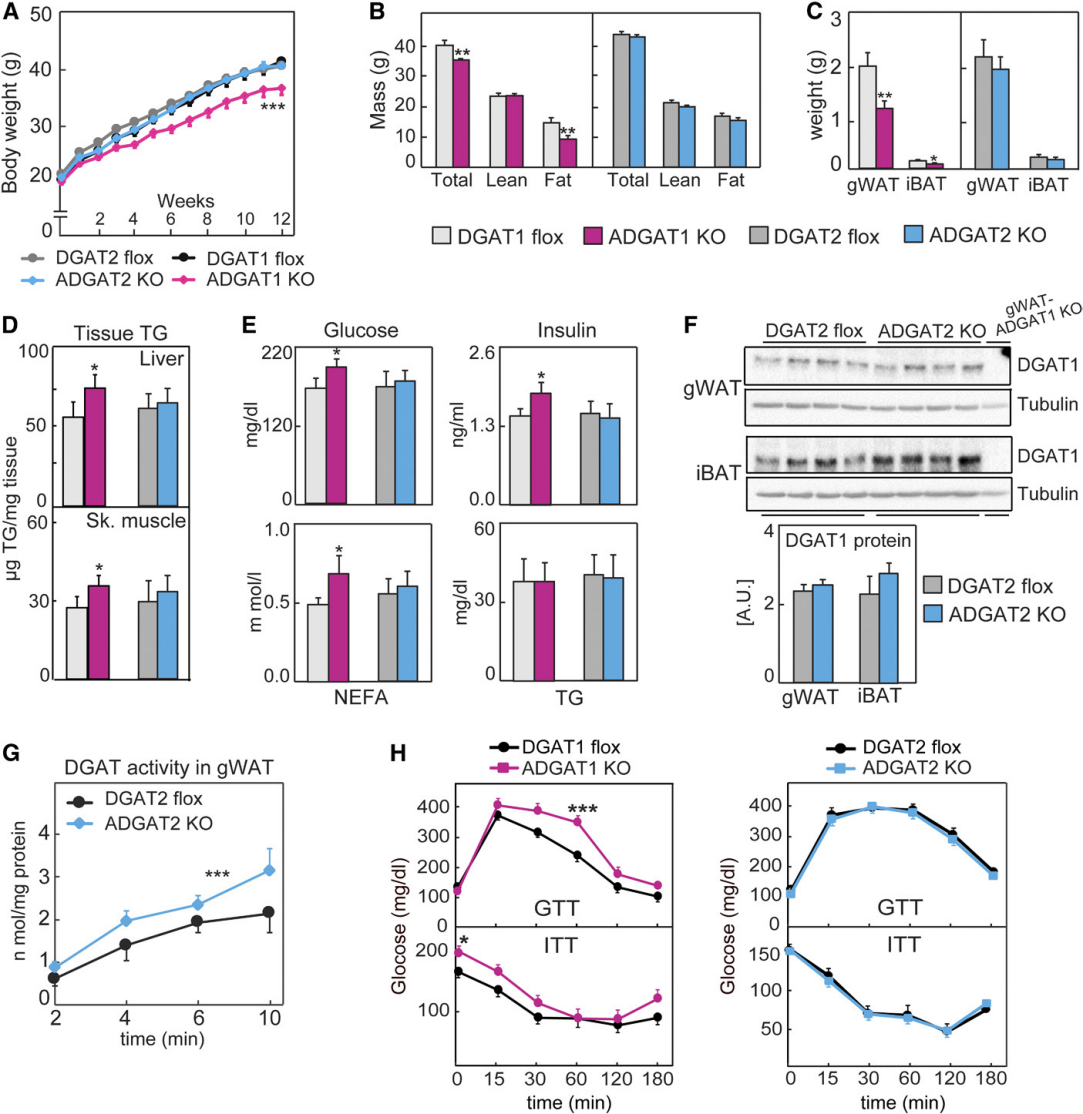
DGAT1 contributes more to diet-induced obesity than DGAT2. A: Body weights of mice fed an HFD (*n* = 15). B: Lean- and fat-mass analysis of mice fed an HFD (*n* = 10 per genotype). C: Weights of gWAT and iBAT of mice fed an HFD (*n* = 8). D: TG content of livers and skeletal muscle (*n* = 6 mice). E: Plasma parameters of mice fed an HFD (*n* = 6). F: Western blot analysis of tissues from mice fed an HFD (*n* = 4). G: Glucose- and insulin-tolerance tests were performed on mice fed an HFD (*n* = 10–16 per genotype). Data are presented as mean ± SD (C–E) or mean ± SEM (A, B, G, H). *P < 0.05, **P < 0.01 by *t*-test, and ***P < 0.001 by two-way ANOVA. iBAT, intrascapular brown adipose tissue; NEFA, nonesterified fatty acid.

To determine the relative contributions of each DGAT enzyme to TG storage during excess fat intake, we fed ADGAT1 KO mice, ADGAT2 KO mice, or control mice a Western-type HFD for 12 (**Fig. 3A**) or 20 (supplemental Fig. S2A, B) weeks. During this time, ADGAT1 KO mice gained ∼10% less body weight than ADGAT2 KO mice or either floxed (control) line of mice, and this reduction in body weight was due to a ∼30% reduction in fat mass, with 40% and 30% reductions in gonadal or brown fat masses, respectively (Fig. 3B, C). Levels of liver and skeletal-muscle TGs were moderately higher in ADGAT1 KO mice fed an HFD than controls (Fig. 3D). With respect to plasma metabolites, ADGAT1 KO mice showed ∼10% increases in plasma levels of glucose, insulin, and free fatty acid levels (Fig. 3E) and impaired glucose and insulin tolerance (Fig. 3H). In contrast, we found no differences in body weight, fat mass, plasma parameters, or glucose or insulin tolerance in ADGAT2 KO mice (Fig. 3A, E). Under these conditions, DGAT1 protein levels in ADGAT2 KO mice were not upregulated (Fig. 3F), indicating a lack of compensation in expression, although the capacity for TG synthesis activity for DGAT1 was increased by ∼25%, suggesting posttranslational regulation (Fig. 3G). For ADGAT1 KO mice, there were no compensatory changes in DGAT2 protein levels.

### HFD feeding causes differential gene-expression changes in mice lacking DGAT1 or DGAT2 in adipose tissue

**Fig. 4.**
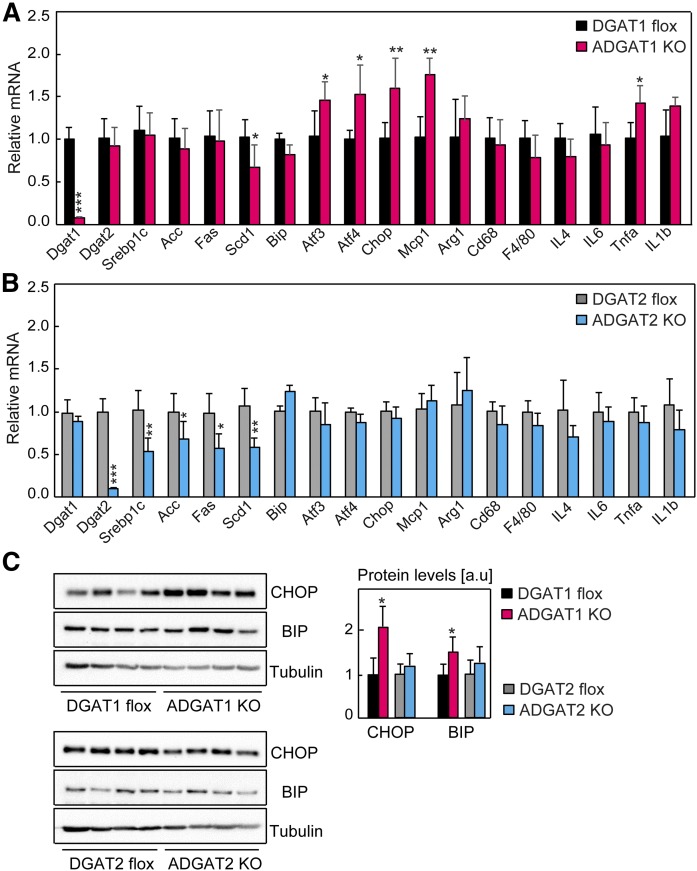
ER stress in ADGAT1 KO mice fed an HFD but not in ADGAT2 KO mice. A, B: mRNA levels of lipogenic genes, ER stress genes, and inflammatory genes determined by real-time qPCR (*n* = 6 mice per genotype). Mice were fed an HFD for 16 weeks. Tissues were collected in the ad libitum-fed state. C: Protein levels of BIP and CHOP in gWAT of mice fed an HFD for 16 weeks (*n* = 4). Data are presented as mean ± SD. *P < 0.05, **P < 0.01, and ***P < 0.001 by *t*-test.

Our findings are consistent with HFD feeding provoking a lipotoxic response when DGAT1 is lacking in adipocytes. We therefore examined the expression of ER stress and inflammatory marker genes in gWAT of ADGAT1 KO mice fed the HFD. We found greater expression of target genes of the unfolded protein response, most notably for the PERK branch of this signaling pathway (*Atf4* and *Chop*) in ADGAT1 KO mice than controls (**Fig. 4A**). Consistent with previous findings that established correlations between unfolded protein response and inflammatory gene expression (39, 40), we found increased expression of the cytokines *Mcp1* and *Tnfα* in gWAT of ADGAT1 KO mice fed an HFD (Fig. 4A). We also found modest increases in the protein levels of ER stress markers BIP and CHOP in gWAT of ADGAT1 KO mice fed an HFD (Fig. 4C). Thus, ADGAT1 KO mice fed an HFD exhibit gene-expression findings consistent with an ongoing lipotoxic response.

In contrast, ADGAT2 KO mice fed the HFD did not exhibit gene-expression changes of lipotoxicity (Fig. 4B), likely because DGAT1 is expressed. Because it has previously been shown that DGAT2 mRNA levels exhibit a positive correlation with the expression of de novo lipogenic genes of the SREBP1 pathway (41, 42), we examined this correlation in adipose tissue of ADGAT2 KO mice. Consistent with previous reports, we found reduced levels of mRNA for *Srebp1c* and numerous *Srebp1c* target genes (Fig. 4B). Such changes were not found in ADGAT1 KO fat, with the exception of a reduction of mRNA levels of *Scd1* (Fig. 4A). Under conditions of an HFD challenge, DGAT1 or DGAT2 inactivation resulted in very different effects with respect to gene expression, with DGAT1 expression being inversely correlated with ER stress and DGAT2 expression being positively correlated with lipogenesis.

## DISCUSSION

By studying murine models lacking either DGAT1 or DGAT2 in adipocytes we can draw several important conclusions with respect to the functions of the two enzymes in TG and energy metabolism. First, and most surprisingly given the previous model that DGAT2 might be the major enzyme responsible for TG synthesis and storage in mice (15, 33), we now find that the two enzymes are functionally redundant with respect to TG storage in animals fed a chow diet. Under basal conditions of chow feeding, the deletion of either enzyme in adipocytes resulted in little to no detectable metabolic phenotype, showing that the enzymes can compensate for each other. This finding is perhaps not totally unexpected given that treatment of many different cell types with inhibitors of either DGAT enzyme does not result in marked reduction of TG synthesis (17, 18). Additionally, these findings are consistent with in vitro analyses of DGAT1 and DGAT2 in mouse embryonic fibroblasts and in 3T3-L1-derived adipocytes, where TG synthesis activity from either enzyme was sufficient to support TG accumulation during differentiation (17, 18). It appears that either DGAT enzyme is fully capable of catalyzing TG synthesis for storage under basal conditions or during adipocyte differentiation.

A second major conclusion was evident when we challenged mice with an HFD. Specifically, only ADGAT1 KO mice were protected (to some degree) from diet-induced obesity. Biochemically, this could be due to preferential channeling of exogenously provided fatty acids to DGAT1 at relatively high substrate concentrations (13, 29, 43). Presumably, DGAT2 is unable to compensate for DGAT1 when high levels of fatty acids are delivered exogenously to cells. For many models, increased leanness is associated with improved glucose and energy metabolism. However, the protection from TG accumulation in ADGAT1 KO mice fed an HFD was accompanied by metabolically detrimental effects consistent with lipotoxicity, including activated ER stress, inflammation, and apparent insulin resistance. That DGAT1-mediated TG synthesis is protective against fatty acid-induced lipotoxicity is becoming abundantly clear. DGAT1 overexpression in several different transgenic mouse models protects these mice from the toxic effects of lipids (41, 43–46). The selective deletion of DGAT1 in several tissues in mice also predisposes these mice to lipotoxicity (47, 48), and humans who lack DGAT1 exhibit dietary fat-induced diarrhea (49). We also found that DGAT1 mRNA levels in human adipose tissue exhibits an inverse correlation with many genes of ER stress (18). All of these studies are consistent with the hypothesis that DGAT1-mediated TG synthesis is lipoprotective for cells and tissues.

Of note, whole-body deletion of DGAT1 in mice paradoxically results in a metabolically protective phenotype rather than one of lipotoxicity (29–31). Two factors likely contribute to this. First, mice express DGAT2 in their small intestine (humans do not) (50), so they are presumably mostly spared from the lipotoxic effects dietary fat could cause in the intestine when DGAT1 is deleted. Second, DGAT1 KO mice exhibit a complex phenotype with the activation of energy expenditure (29, 30), including increased fatty acid oxidation (51), that likely prevents the accumulation of lipids in tissues. This phenotype includes complex metabolic effects that result in improved insulin and leptin sensitivity (30) for reasons that are still poorly understood but may result from changes in secreted factors (52).

We previously showed that DGAT2 is essential for mice (33). We now provide evidence, through an epidermis-specific deletion, that one essential function for the enzyme is a cell type-autonomous, crucial role in maintaining the transepidermal water barrier. How DGAT2 contributes to the barrier is currently unclear. One possibility is that the enzyme is required for the production of specific ω-*O*-acylceramide levels that are thought to be crucial for the lipids of the corneocyte envelope involved in forming the water barrier (53) and that are reduced in the skin of DGAT2 KO mice (33). In addition to DGAT2, the loss of other lipid metabolism genes in murine skin also results in a similar phenotype (53–55), indicating a complex and crucial pathway of lipid metabolism in this cell type. With respect to DGAT2, the enzyme may either directly contribute to the synthesis of these lipids or function indirectly by enabling the storage of precursors for their synthesis. Humans with DGAT2 deficiency appear not to have such marked skin abnormalities, although DGAT2 function has been linked to psoriasis (56).

Finally, our study sheds additional light on DGAT2 function. We show that DGAT2 can function to esterify fatty acids generally if concentrations are not too high, and there is an emerging connection of DGAT2 with de novo lipogenesis. This includes several studies that have linked DGAT2 activity to de novo synthesis of fatty acids (43, 57) and this and other studies (42, 58) that suggest a feedback mechanism correlating DGAT2 activity with the SREBP1-mediated pathway. Notably, DGAT2 is evolutionarily conserved as a major TG-synthesis enzyme across eukaryotes (15, 16), whereas DGAT1 is a member of the MBOAT gene family, several of whose lipid-esterifying members have been linked to the detoxification of lipids in the ER (59). Thus, collectively, a picture emerges in which DGAT2 has a more ancient function for mediating TG synthesis of de novo-synthesized fatty acids, and DGAT1, which can utilize a variety of acyl acceptor substrates (15, 24), has a specific function in ER protection.

## Supplementary Material

Supplemental Data
