# Supplementary material for: The triglyceride synthesis enzymes DGAT1 and DGAT2 have distinct and overlapping functions in adipocytes
Source: J Lipid Res. 2019 Apr 1;60(6):1112–20. doi: 10.1194/jlr.M093112 (PMC6547635; doi:10.1194/jlr.M093112)
Supplement: Supplemental Data [file supp_60_6_1112__index.html]

The triglyceride synthesis enzymes DGAT1 and DGAT2 have distinct and overlapping functions in adipocytes — The triglyceride synthesis enzymes DGAT1 and DGAT2 have distinct and overlapping functions in adipocytes — Supplemental Data 

# The triglyceride synthesis enzymes DGAT1 and DGAT2 have distinct and overlapping functions in adipocytes

## Supplemental Data

- Supplemental Figure 1 (.pdf, 1.2 MB) - Validation of founders for gene-trap allele, and DGAT2 flox mice by PCR.
- Supplemental Figure 2 (.pdf, 43 KB) - ADGAT1 KO mice are resistant to HFD induced weight gain.
