# Supplementary material for: The triglyceride synthesis enzymes DGAT1 and DGAT2 have distinct and overlapping functions in adipocytes
Source: J Lipid Res. 2019 Apr 1;60(6):1112–20. doi: 10.1194/jlr.M093112 (PMC6547635; doi:10.1194/jlr.M093112)
Supplement: Supplemental Data [file 10.1194_M093112_jlr.M093112-1.pdf]

Supplemental Figure S1

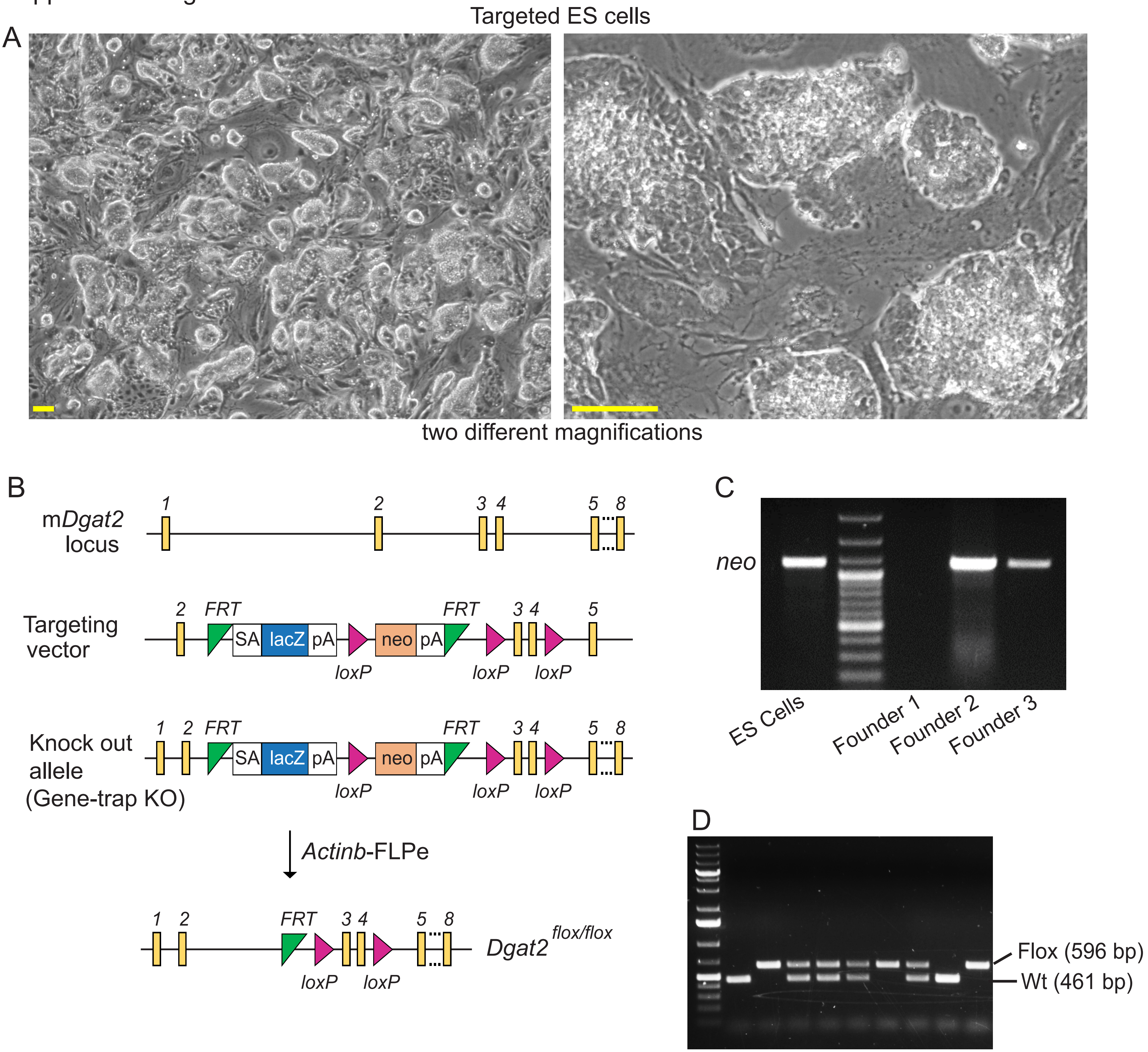

Fig. S1. Validation of founders for gene-trap allele, and DGAT2 flox mice by PCR. (*A*) Targeted ES-cells used for blastocyst injection. (*B*) Strategy for generating *Dgat2* gene-trap knockout mice, and DGAT2 flox mice. (*C*) Genotyping of founders for gene-trap allele. (*D*) Genotyping of DGAT2 flox mice.
