# Supplementary material for: The triglyceride synthesis enzymes DGAT1 and DGAT2 have distinct and overlapping functions in adipocytes
Source: J Lipid Res. 2019 Apr 1;60(6):1112–20. doi: 10.1194/jlr.M093112 (PMC6547635; doi:10.1194/jlr.M093112)
Supplement: Supplemental Data [file 10.1194_M093112_jlr.M093112-2.pdf]

Supplemental Figure S2

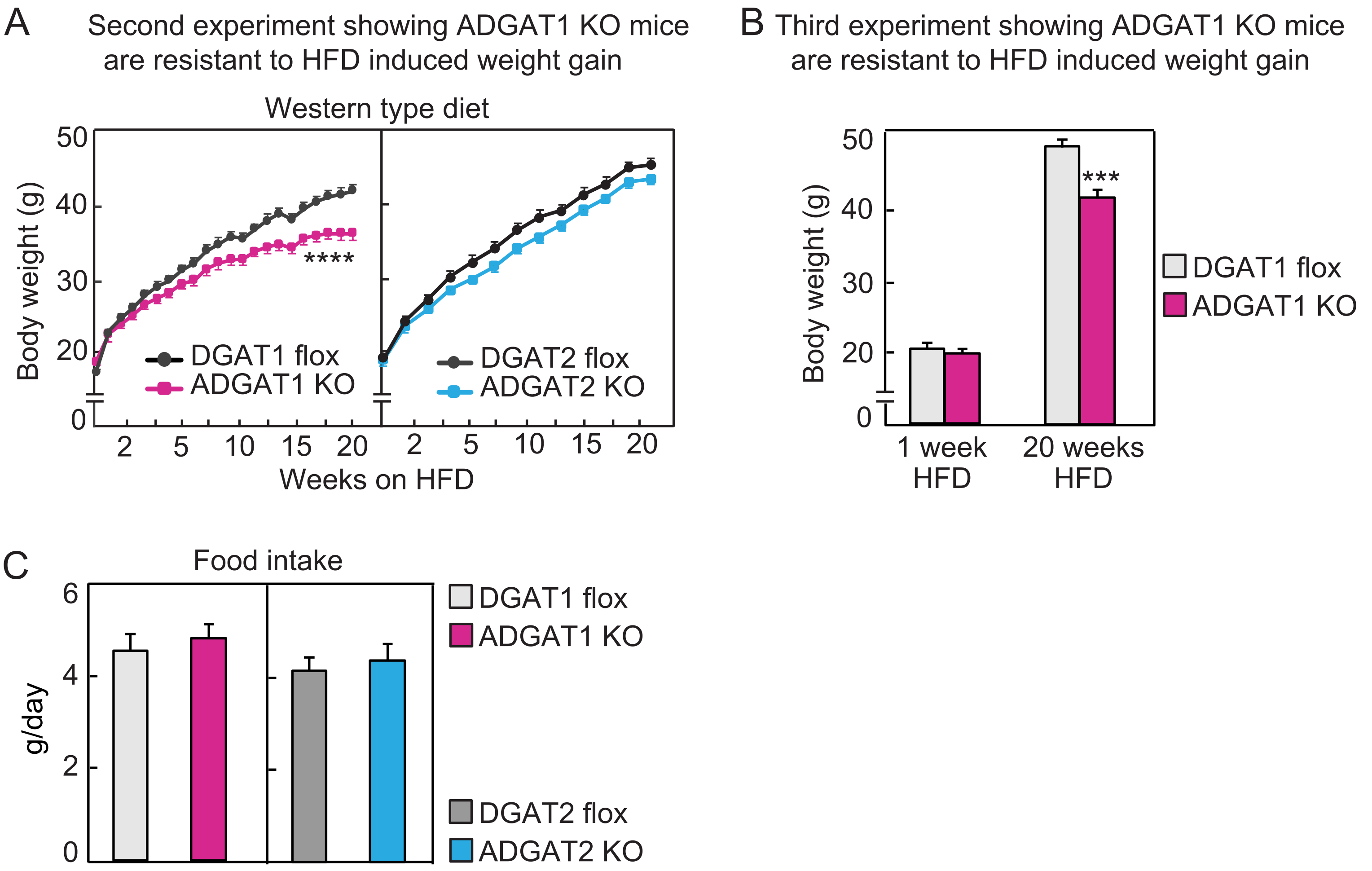

Fig. S2. ADGAT1 KO mice are resistant to HFD induced weight gain. (*A*) Body weights of HFD-fed mice (n=12 for ADGAT1 KO, n=20 for ADGAT2 KO). (*B*) Body weights of HFD-fed mice (n=12). (*C*) Daily food intake (n=4 mice per genotype). Data are presented as mean ± SD (*C*), as mean ± SEM (*A* and *B*). ***p<0.001 by *t*-test; ****p<0.001 by two-way ANOVA.
